# On Diseases of the Mouth in Infancy and Childhood

**Published:** 1859-07

**Authors:** William G. Smull


### ARTICLE II.

### *On Diseases of the Mouth in Infancy and Childhood.*

BY WILLIAM G. SMULL, M. D.

### APHTHA.

THERE are but few organs whose diseases maintain such constant relation to any period of life, as diseases of the mouth do to that of childhood. It might be almost asserted as an impossibility, that infancy could pass through the period ending with dentition, without suffering from some of the various diseases of the mouth, peculiar to that age. The physiological cause of this association of infancy and disease, must be found in the fact of the great activity with which the vital processes are carried on, and their consequent liability to derangement. Secretion is active; the membranes are exceedingly vascular; and the mucus is copiously poured out upon the mucous membranes of the digestive organs. We may suppose then, that any cause that would alter the quantity, or impair the quality of the secretions, or that would disturb vascular action, would be promptly felt by the general system, and produce changes in organs more or less remote from the primary source of disease.

Diseases of the mouth of children have been divided into idiopathic and symptomatic; but why should we suppose that they are not altogether symptomatic? It is true, that we cannot arrive at a positive knowledge of the state of the other organs, but it would be fair reasoning to infer that the stomach is always the organ primarily at fault. This inference is supported by the fact well known, that where children are deprived of the mother's milk, some of the various forms of disease of the mouth are almost invariable consequences. Again, when from some change in the secretions of the stomach, or from a want of power in that organ, the ingesta become acid, the mucous membrane of the mouth at once indicates by its altered appearance, the degree of deviation from healthy action that exists in the stomach. This conclusion is not altered by the fact, that children who nurse are likewise frequently the subjects of affections of the mouth ; for the variation in the chemical ingredients of the mother's milk, will often produce consequences more grave than disease of the mucous membranes, and may prove fatal to the infant.

The primary causes of diseases of the mouth in children are somewhat irrelevant to the purpose of this article; they are alluded to for the reason that the treatment of these affections will bring us back again to the cause, which has to be removed first, by remedial and hygeinic measures.

No little confusion prevails among authors with reference to the nomenclature and pathology of this class of diseases. For our purpose it will be sufficient to inquire into the varieties of disease affecting the mouth, and guided by the light of increased knowledge of pathology, to suggest names suited to morbid conditions. It is not our desire to add new names to diseases long known, but to select such as have been used, but have been applied improperly to different pathological conditions.

The forms of diseases of the mouth, clearly recognizable, are as follows :

Aphtha, follicular stomatitis, ulcerative stomatitis, and gangrenous stomatitis or cancrum oris. One classification progresses from the milder to the graver forms and our notice of them will follow the same sequence.

The first, and the most common of these is aphtha. This affection is variously known as aphtha infantum, milk-thrush and *muguet*. It makes its appearance as white specks or curdy looking vesicles upon the tongue and mucous membrane of cheeks, palate, and posterior fauces. Although its name would indicate it to be of an inflammatory origin, yet it is not characterized by any appearance of inflammation. These white flakes gradually fall off and leave the cutis vera unbroken but abraided. The pathology of this disease is not yet decided upon. It is supposed by some to be a vesicular eruption, similar to those which take place elsewhere upon the body. By some it is considered to be a diphthritic deposit, formed in the same manner that false membranes are produced, and others again regard it as the result of a peculiar parasitic growth, similar in its production to *porrigo*. Whatever may be its pathology the microscope shows parasitic growths to be a part of its character.

Dr. J. H. Bennett states, that epithelial scales often lose their transparency, become opaque, and give rise to confervoid growths, and that "in infants the tongue and cavity of the mouth are not unfrequently covered with a yellowish flocculent matter, constituting the disease named *muguet* by the French, in which sporules and confervoid growths in a high state of development, may be detected in considerable numbers." The presence then of confervæ may be the source of the disease, or it may be an accidental development taking place upon a diseased membrane. It may be the cause or the effect of disease: their existence is important and may lead future investigators to important results in the pathology and treatment of diseases of the mouth.

Leaving the pathology of this disease as an open question, we will trace its progress to its termination. We seldom find that aphtha disappears entirely after the appearance of a few patches of the eruption: but it shows itself in other parts of the mouth and fauces; the patches sometimes fading in one place to be renewed in another. Sometimes they are distinct and at others coalesce: at times the entire mucous membrane of the mouth is covered with it. It cannot be supposed that under such circumstances, the delicately organized system of the infant should escape feeling the morbid state of its mouth. Fever, diarrhea and restlessness, are its usual accompaniments, and where the symptoms do not mitigate, the little patient sinks, under the irritative and exhausting processes. In fatal cases of aphtha, post mortem examination shows the mucous membrane of the intestinal canal to be affected in the same manner that that of the mouth is, and cases are mentioned where the entire intestinal mucous membrane had an aphthous appearance.

We will make a hasty summary of the treatment of aphtha. The predisposing causes first demand our attention; the fault may be in the character of the nourishment the child receives—nothing can adequately supply the place of the mother's milk, and should there be any fault here, our treatment should be partly addressed to the mother. The state of the child's stomach should be ascertained; acidity, and evacuations of greenish substances, indicate small doses of mercurials and antacids. Our subsequent treatment should be some of the preparations of potash, (of which the chlorate is preferable) soda, &c. A variety of mouth washes have popularity among physicians and nurses, among them perhaps a simple solution of borax would be the most efficacious: occasionally astringent and slightly stimulating washes change the condition of the mouth, among these are infusions of rose leaves, sage, &c. Where the disease assumes a still graver form, our treatment must be the internal use of tonics and stimulants, with washes of the solution of the nitrate of silver and other antiseptic applications.

### ARTICLE III.

*Alveolar Abscess.* By H. N. WADSWORTH, D. D. S.

